# Prognostic significance of inflammatory response markers for locally advanced squamous cell carcinoma of the external auditory canal and middle ear

**DOI:** 10.1093/jrr/rrab048

**Published:** 2021-06-14

**Authors:** Kenji Makita, Yasushi Hamamoto, Noriko Takata, Hirofumi Ishikawa, Shintaro Tsuruoka, Kotaro Uwatsu, Naohito Hato, Teruhito Kido

**Affiliations:** Department of Radiology, Ehime University Graduate School of Medicine, 454 Shitsukawa, Toon, Ehime 791-0295, Japan; Departments of Radiation Oncology, National Hospital Organization Shikoku Cancer Center, Kou-160, Minami-Umenomoto-Machi, Matsuyama, Ehime 791-0280, Japan; Departments of Radiation Oncology, National Hospital Organization Shikoku Cancer Center, Kou-160, Minami-Umenomoto-Machi, Matsuyama, Ehime 791-0280, Japan; Department of Radiology, Ehime University Graduate School of Medicine, 454 Shitsukawa, Toon, Ehime 791-0295, Japan; Department of Radiology, Ehime University Graduate School of Medicine, 454 Shitsukawa, Toon, Ehime 791-0295, Japan; Department of Radiology, Ehime University Graduate School of Medicine, 454 Shitsukawa, Toon, Ehime 791-0295, Japan; Department of Radiology, Ehime University Graduate School of Medicine, 454 Shitsukawa, Toon, Ehime 791-0295, Japan; Department of Otorhinolaryngology, Head and Neck Surgery, Ehime University Graduate School of Medicine, 454 Shitsukawa, Toon, Ehime 791-0295, Japan; Department of Radiology, Ehime University Graduate School of Medicine, 454 Shitsukawa, Toon, Ehime 791-0295, Japan

**Keywords:** squamous cell carcinoma (SCC), external auditory canal (EAC), middle ear (ME), radiotherapy, inflammatory response marker, C-reactive protein-to-albumin ratio

## Abstract

We investigated the prognostic significance and treatment outcomes of pretreatment inflammatory response markers for locally advanced squamous cell carcinoma (SCC) of the external auditory canal (EAC) and middle ear (ME). Between July 2003 and July 2019, 21 patients with SCC of the EAC (n = 18) or ME (n = 3) who received radiotherapy with or without surgery or systemic therapy (radiotherapy alone [n = 2], radiotherapy + systemic therapy [n = 6], radiotherapy + surgery [n = 7], radiotherapy + surgery + systemic therapy [n = 6]) were retrospectively examined. The median radiation dose was 66.0 (range, 50.4–70.0) Gy, with daily fractions of 1.8–2.0 Gy. The median follow-up period was 25 months (range, 6–137). The two-year overall survival (OS), progression-free survival (PFS), and locoregional control (LC) rates were 61%, 48%, and 55%, respectively. OS, PFS, and LC did not differ significantly according to patient- (age, sex), tumor- (Pittsburgh stage, pretreatment neurological findings), and treatment-related (surgery or systemic therapy, radiation dose, prophylactic neck irradiation) factors. Conversely, there were significant differences in OS, PFS, and LC between patients with high and low pretreatment C-reactive protein-to-albumin ratios (p = 0.002, 0.003, and 0.004, respectively). OS also differed significantly between patients with high and low pretreatment neutrophil-to-lymphocyte ratios (NLR; p = 0.037). Other inflammatory response markers, including platelet-to-lymphocyte ratio (PLR) and albumin-to-globulin ratio (AGR), did not influence OS, PFS, or LC. Our findings suggest that pretreatment C-reactive protein-to-albumin ratio and NLRs have a significant impact on treatment outcomes in patients with locally advanced SCC of the EAC and ME.

## INTRODUCTION

Squamous cell carcinoma (SCC) of the external auditory canal (EAC) and middle ear (ME) is a rare disease, with a reported prevalence of approximately one per million individuals [1, 2]. Although several treatment options have been proposed (surgery with or without postoperative chemoradiotherapy and definitive radiotherapy with or without chemotherapy), the optimal therapeutic strategy has not yet been established. Complete resection is usually recommended for SCC of the EAC and ME [3, 4].

Studies [5, 6] have reported an association between the host inflammatory response and cancer growth. Cancer progression requires interactions between cancer cells and their microenvironment. Systemic inflammatory response is associated with tumor microenvironment. It promotes microvascular regression and differentiation of cancer cells and suppresses the activity of host immune cells [7–11]. Thus, systemic inflammatory response may support tumor progression. Previous studies [12–14] have examined the prognostic significance of inflammatory response markers, including neutrophil-to-lymphocyte ratio (NLR), C-reactive protein (CRP)-to-albumin ratio (CAR), platelet-to-lymphocyte ratio (PLR), and albumin-to-globulin ratio (AGR), for various cancers. Recently, Li *et al.* [15] showed that preoperative NLR was significantly correlated with tumor recurrence in patients with SCC of the EAC. The authors concluded that preoperative NLR may be an unfavorable prognostic factor for SCC of the EAC. However, they analyzed the early Pittsburgh stage [16] without evaluating treatment outcomes according to the Pittsburgh stage. In this study, we aimed to evaluate the prognostic significance of pretreatment inflammatory response markers in patients with locally advanced SCC of the EAC or ME who received definitive or adjuvant radiotherapy.

## MATERIALS AND METHODS

### Ethical approval

The study design was approved by the appropriate ethical committee (approval number: 2010001), and all participants provided informed consent.

### Patients

Between July 2003 and July 2019, 24 patients with locally advanced SCC of the EAC or ME who received radiotherapy with or without surgery or systemic therapy as initial treatment were retrospectively examined. Pretreatment NLR, CAR, PLR, and AGR data were available for 21 patients (male [n = 8], female [n = 13]) who were included in the analysis. The median age was 65 (range, 41–83) years. Eighteen patients had SCC of the EAC, and three patients had SSC of the ME. One patient had neck node metastases. None of the patients had distant metastases at presentation.

Pretreatment laboratory tests were usually performed within 10 (median, six [range, 1–18]) days before the start of initial treatment. All patients were diagnosed and staged based on findings of physical examination, computed tomography, and/or magnetic resonance imaging at presentation. These modalities were used in follow-up visits after the initial treatment to detect locoregional recurrence or distant metastases. Locoregional recurrence was defined as tumor regrowth in local and neck nodes. Progression-free survival (PFS) was defined as the time after treatment without deterioration of symptoms and disease, local regrowth, or appearance of metastatic lesions within or outside the irradiated field.

### Treatments

Treatment strategies were determined based on the Pittsburgh staging system [16] and patients’ general condition. Seven patients received radiotherapy and surgery; six received radiotherapy, surgery, and systemic therapy; six received radiotherapy and systemic therapy; and two received radiotherapy alone. The median radiation doses for the definitive radiotherapy with or without systemic therapy regimen and radiotherapy and surgery with or without systemic therapy regimen were 66.0 (range, 66.0–70.0) and 66.0 (range, 50.4–70.0) Gy, respectively. Twelve patients received local irradiation and nine received local and prophylactic neck irradiation. In principle, radiotherapy and systemic therapy consisted of cisplatin (80 mg/m2 on day one every three weeks). Ten patients received fluoropyrimidine-based chemotherapy (tegafur/gimeracil/oteracil/potassium [n = 3], tegafur/uracil [n = 2], and 5-fluorouracil [n = 5]) with or without cisplatin. In addition, two patients received cetuximab after radiotherapy.

### Statistical analyses

Survival rates were calculated from the start of initial treatment using the Kaplan–Meier method. The log-rank test was used to evaluate differences in overall survival (OS), PFS, and locoregional control (LC). Pretreatment NLR, CAR, PLR, and AGR values were calculated. There are no established cutoff values for NLR, CAR, PLR, and AGR in SCC of the EAC and ME. To determine the optimal cutoff values for predicting OS, PFS, and LC in patients with SCC of the EAC and ME, receiver operating characteristic (ROC) curve analysis was performed. The areas under the ROC curves for OS and PFS were 0.62 (sensitivity, 90%; specificity, 45%), 0.70 (sensitivity, 55%; specificity, 65%), 0.55 (sensitivity, 70%; specificity, 55%), and 0.62 (sensitivity, 60%; specificity, 73%) for NLR, CAR, PLR, and AGR, respectively. The areas under the ROC curves for LC were 0.54 (sensitivity, 100%; specificity, 25%), 0.59 (sensitivity, 44%; specificity, 98%), 0.53 (sensitivity, 56%; specificity, 67%), and 0.56 (sensitivity, 89%; specificity, 33%) for NLR, CAR, PLR, and AGR, respectively. For OS and PFS, an NLR of 3.95, CAR of 0.31, PLR of 216, and AGR of 1.34 corresponded to the maximum sum of sensitivity and specificity. For LC, an NLR of 4.43, CAR of 0.31, PLR of 135.9, and AGR of 1.40 corresponded to the maximum sum of sensitivity and specificity. Statistical analyses were conducted using JMP software, version 14.3.0 (SAS Institute, Cary, NC, USA).

## RESULTS

**Fig. 1.**
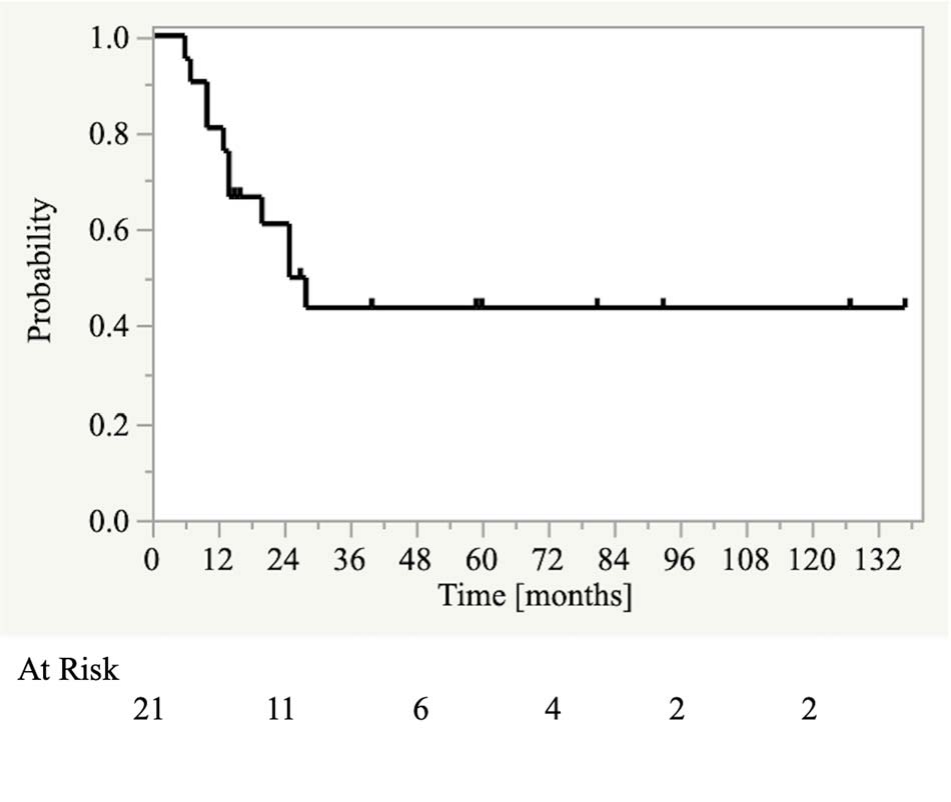
Kaplan–Meier curves for overall survival.

**Fig. 2.**
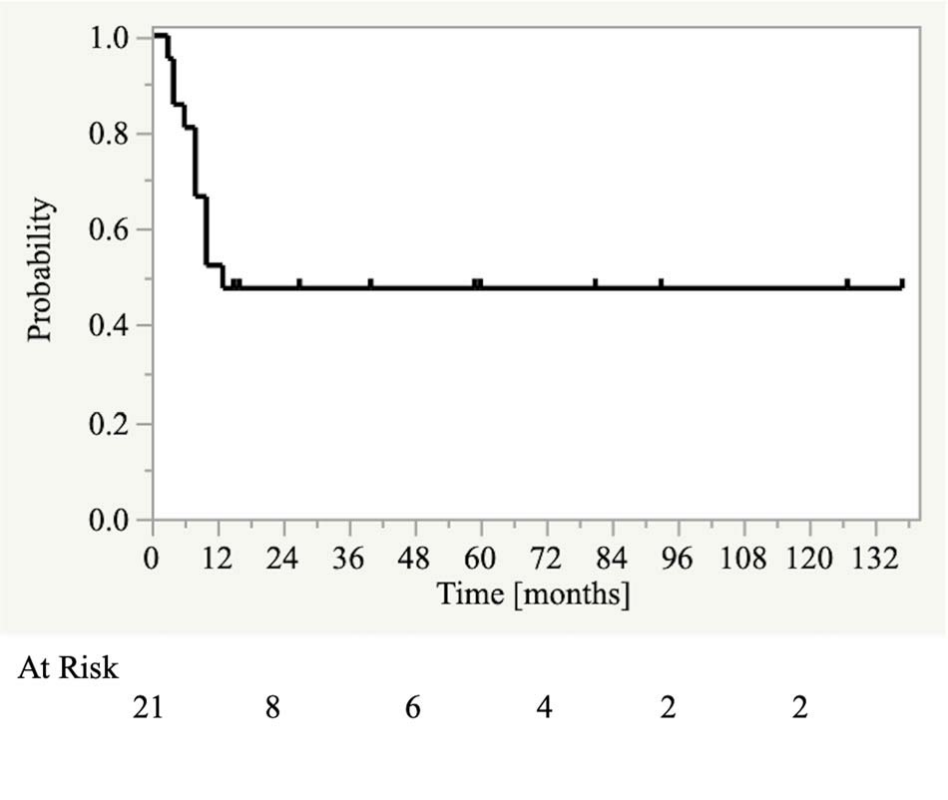
Kaplan–Meier curves for progression-free survival.

Patients’ characteristics are presented in Table 1. The median follow-up period was 25 (range, 6–137) months. The one- and two-year OS (Fig. 1), PFS (Fig. 2), and LC rates were 81% and 61%, 52%, 48%, 68%, and 55%, respectively. Nine patients experienced tumor recurrence. All nine patients had in-field recurrences. Five patients had in-field recurrence alone, three had in-field recurrence and regional lymph node metastasis outside the radiation field, and one had in-field recurrence and distant metastasis. One patient with neck node metastases who was treated with concurrent chemoradiotherapy had no recurrence or distant metastasis at the last follow-up.

**Table 1:** Patient characteristics

Characteristic		Value
No. of patients		21
Age		median 65 (41–83)
	< 65 years	10 (47.6%)
	≥ 65 years	11 (52.4%)
Aex	male	8 (38.1%)
	female	13 (61.9%)
PS (ECOG)	0	17 (80.9%)
	1	3 (14.3%)
	3	1 (4.8%)
Pittsburgh staging	T3	10 (47.6%)
	T4	11 (52.4%)
Neurological findings before treatment	yes	8 (38.1%)
	no	13 (61.9%)
Surgery	yes	13 (61.9%)
	subtotal resection	10 (47.6%)
	complete resection	3 (14.3%)
	no	8 (38.1%)
Systemic therapy	yes	10 (47.6%)
	no	11 (52.4%)
Radiation dose (Gy)		median 66 (50.4–70.0)
	< 66	4 (19.0%)
	≥ 66	17 (81.0%)
Irradiated field	local	12 (57.1%)
	local + prophylactic	9 (42.9%)
NLR		median 2.33 (1.02–12.9)
	< 3.95	16 (76.2%)
	≥ 3.95	5 (23.8%)
CAR		median 0.04 (0.002–0.84)
	< 0.31	16 (71.4%)
	≥ 0.31	5 (28.6%)
PLR		median 153.1 (41.8–504.9)
	< 216	12 (57.1%)
	≥ 216	9 (42.9%)
AGR		median 1.32 (0.88–2.0)
	< 1.34	11 (52.4%)
	≥ 1.34	10 (47.6%)

EAC; external auditory canal, ME; middle ear, PS (ECOG); performance status (Eastern Cooperative Oncology Group), NLR; neutrophil-to-lymphocyte ratio, CAR; C-reactive protein-to-albumin ratio, PLR; platelet-to-lymphocyte ratio, AGR; albumin-to-globulin ratio.

Values are number (percentage) or median (range).

**Fig. 3.**
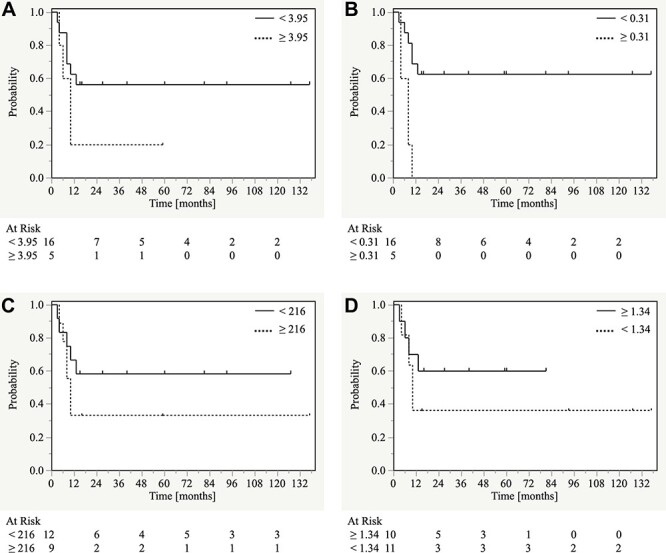
Kaplan–Meier curves for progression-free survival according to pretreatment inflammatory response markers. (A) Neutrophil-to-lymphocyte ratio (<3.95 vs ≥3.95), (B) C-reactive protein-to-albumin ratio (<0.31 vs ≥0.31), (C) platelet-to-lymphocyte ratio (<216 vs ≥216), and (D) albumin-to-globulin ratio (<1.34 vs ≥1.34)

### Survival and LC rates stratified by Pretreatment NLR

The one-year OS rate was significantly different between patients with NLR <3.95 and those with NLR ≥3.95 (94% vs 40%; p = 0.037). Conversely, the one-year PFS rate was not significantly different between the two groups (63% vs 20%; p = 0.160) (Fig. 3a and Table 2). There was also no significant difference in the one-year LC rate between patients with NLR <4.43 and those with NLR ≥4.43 (69% vs 75%; p = 0.902) (Table 3).

**Table 2:** Univariate analysis of overall survival and progression-free survival rates

Variables		OS			DFS		
		One-year (%)	Two-year (%)	p	One-year (%)	Two-year (%)	p
Age	< 65 years (n = 10)	80	58	0.9737	50	50	0.7912
	≥ 65 years (n = 11)	82	64		55	45	
Sex	male (n = 8)	75	63	0.7274	50	38	0.4961
	female (n = 13)	85	59		54	54	
Pittsburgh staging	T3 (n = 10)	80	70	0.4986	70	60	0.2486
	T4 (n = 11)	82	55		36	36	
Neurological findings before treatment	yes (n = 8)	88	63	0.8889	50	50	0.8762
	no (n = 13)	77	61		54	46	
Surgery	yes (n = 13)	85	61	0.9332	46	46	0.8117
	no (n = 8)	75	63		63	50	
Systemic therapy	yes (n = 9)	78	65	0.8968	44	44	0.8652
	no (n = 12)	83	58		58	50	
Radiation dose (Gy)	< 66 Gy (n = 4)	75	-	0.0747	25	-	0.3079
	≥ 66 Gy (n = 17)	82	70		59	53	
Irradiated field	local (n = 12)	83	58	0.1593	42	33	0.1209
	local + prophylactic (n = 9)	78	67		67	67	
NLR	< 3.95 (n = 16)	94	74	0.0365	63	56	0.1603
	≥ 3.95 (n = 5)	40	20		20	20	
CAR	< 0.31 (n = 16)	88	74	0.0019	69	63	0.0025
	≥ 0.31 (n = 5)	60	20		0	0	
PLR	< 216 (n = 12)	92	74	0.129	67	58	0.2853
	≥ 216 (n = 9)	67	44		33	33	
AGR	< 1.34 (n = 11)	73	44	0.1993	36	36	0.3666
	≥ 1.34 (n = 10)	90	80		70	60	

OS; overall survival, PFS; progression-free survival, NLR; neutrophil-to-lymphocyte ratio, CAR; C-reactive protein-to-albumin ratio, PLR; platelet-to-lymphocyte ratio, AGR; albumin-to-globulin ratio.

**Table 3:** Univariate analysis of locoregional control rates

Variables		LC		
		One-year (%)	Two-year (%)	p
Age	< 65 years (n = 10)	79	53	0.5625
	≥ 65 years (n = 11)	58	58	
Sex	male (n = 8)	75	60	0.9379
	female (n = 13)	64	51	
Pittsburgh staging	T3 (n = 10)	79	79	0.1862
	T4 (n = 11)	57	34	
Neurological findings before treatment	yes (n = 8)	58	58	0.6727
	no (n = 13)	75	54	
Surgery	yes (n = 13)	65	43	0.7230
	no (n = 8)	75	75	
Systemic therapy	yes (n = 9)	65	65	0.7374
	no (n = 12)	73	36	
Radiation dose (Gy)	< 66 Gy (n = 4)	38	-	0.3707
	≥ 66 Gy (n = 17)	74	60	
Irradiated field	local (n = 12)	52	35	0.0560
	local + prophylactic (n = 9)	89	76	
NLR	< 4.43 (n = 17)	69	53	0.9018
	≥ 4.43 (n = 4)	75	75	
CAR	< 0.31 (n = 16)	87	69	0.0035
	≥ 0.31 (n = 5)	0	0	
PLR	< 135.9 (n = 8)	88	58	0.8190
	≥ 135.9 (n = 13)	53	53	
AGR	< 1.40 (n = 13)	59	39	0.0792
	≥ 1.40 (n = 8)	86	86	

LC; locoregional control, NLR; neutrophil-to-lymphocyte ratio, CAR; C-reactive protein-to-albumin ratio, PLR; platelet-to-lymphocyte ratio, AGR; albumin-to-globulin ratio.

### Survival and LC rates stratified by Pretreatment CAR

The one-year OS rate was significantly different between patients with CAR <0.31 and those with CAR ≥0.31 (88% vs 60%; p = 0.002). The one-year PFS rate was also significantly different between the two groups (69% vs 0%; p = 0.003) (Fig. 3b and Table 2). Furthermore, there was a significant difference in the one-year LC rate between patients with CAR <0.31 and those with CAR ≥0.31 (87% vs 0%; p = 0.004) (Table 3).

### Survival and LC rates stratified by Pretreatment PLR

The one-year OS rate was not significantly different between patients with PLR <216 and those with PLR ≥216 (92% vs 67%; p = 0.129). The one-year PFS rate was also not significantly different between the two groups (67% vs 33%; p = 0.285) (Fig. 3c and Table 2). Furthermore, there was no significant difference in the one-year LC rate between patients with PLR <135.9 and those with PLR ≥135.9 (88% vs 53%; p = 0.819) (Table 3).

### Survival and LC rates stratified by pretreatment AGR

The one-year OS rate was not significantly different between patients with AGR <1.34 and those with AGR ≥1.34 (73% vs 90%; p = 0.199). The one-year PFS rate was also not significantly different between the two groups (36% vs 70%; p = 0.367) (Fig. 3d and Table 2). Furthermore, there was no significant difference in the one-year LC rate between patients with AGR <1.40 and those with AGR ≥1.40 (59% vs 86%; p = 0.079) (Table 3).

### Survival and LC rates according to patient-, tumor-, and treatment-related factors

OS and PFS did not differ significantly according to age (<65 vs ≥65 years), sex, Pittsburgh stage (T3 vs T4), radiation dose (<66.0 vs ≥66.0 Gy), irradiated fields (with or without prophylactic neck irradiation), use of surgery, and addition of systemic therapy (Table 2). Furthermore, there were no significant differences in LC according to age, sex, Pittsburgh stage, radiation dose, irradiated fields, use of surgery, and addition of systemic therapy (p = 0.563, 0.938, 0.186, 0.371, 0.056, 0.723, and 0.737, respectively) (Table 3).

## DISCUSSION

In this study, the two-year OS and PFS rates for patients with locally advanced SCC of the EAC and ME were approximately 60% and 50%, respectively. The OS and PFS curves plateaued by two and one years from the start of treatment, respectively. There were no significant differences in OS or PFS rates according to patient-, tumor-, and treatment-related factors. However, there were significant differences in both OS and PFS between the high and low CAR groups. Similarly, there was a significant difference in OS between the high and low NLR groups.

Chronic inflammation in the tumor microenvironment can promote malignant tumor progression [17]. In this study, pretreatment CAR was significantly associated with OS and PFS in patients with locally advanced SCC of the EAC and ME. A previous meta-analysis [4] showed that a high CAR is associated with a relatively poor outcome in patients with solid tumors, including those with head and neck tumors. However, the relationship between CAR and treatment outcomes in patients with locally advanced SCC of the EAC and ME has not been well-documented. Serum CRP and albumin levels can be measured in peripheral blood samples. CRP is produced by hepatocytes as a systemic response to cytokines, particularly interleukin-6. It is released from leukocytes within the tumor microenvironment and has been associated with progressive disease and relatively poor survival in patients with different types of cancer [18–20]. In addition, inflammation is associated with decreased serum albumin levels owing to suppressed liver function, resulting in reduced albumin production. The release of cytokines from inflammatory cells may increase microvascular permeability and increase the flow of serum albumin into the extravascular compartment [21]. These findings suggest that CAR is useful for assessing the extent of inflammation in the tumor microenvironment and predicting prognosis. The present findings indicate that CAR is associated with treatment outcomes in patients with locally advanced SCC of the EAC and ME. Overall, this evidence suggests that prognostication of locally advanced SCC of the EAC and ME requires the assessment of tumor- and treatment-related factors, as well as those related to the tumor microenvironment.

In this study, there was no significant difference in PFS between the high and low pretreatment NLR, PLR, and AGR groups. However, a low NLR and PLR and a high AGR were associated with improved PFS. A previous meta-analysis [22] showed that a high NLR was associated with a poor outcome in patients with SCC of the head and neck. In this study, significant differences in OS, but not PFS, were observed between patients with a high and low NLR. Therefore, NLR may be relevant for the prognostication of locally advanced SCC of the EAC and ME. Previous studies [23, 24] have shown that pretreatment PLR and AGR affect prognosis of several types of cancer. However, the role of these markers in the present context remains unclear and requires further validation.

A previous study [25] showed that definitive chemoradiotherapy may achieve comparable outcomes to surgical resection with or without radiotherapy. In this study, no significant differences in outcomes were observed between patients who did and did not undergo surgery. When complete resection is difficult, definitive chemoradiotherapy may improve outcomes of patients with locally advanced SCC of the EAC and ME. However, in this study, the chemotherapy and combination therapy regimens were heterogeneous and the sample size was small; thus, further studies are required to determine the effectiveness of chemoradiotherapy.

This study had some limitations associated with its retrospective nature. First, the sample size was small. Although previous studies have reported significant associations between several prognostic factors and outcomes of patients with SCC of the EAC and ME [3, 16, 26, 27], this study did not replicate these findings, likely because of the small sample size. However, an association between pretreatment CAR and outcomes was observed, despite the small sample size. Consequently, we believe that pretreatment CAR may be useful for prognostication. Nevertheless, further studies are required to validate this association in locally advanced SCC of the EAC and ME. Second, this study included patients undergoing different types of treatment. As there are no standard treatments for locally advanced SCC of the EAC and ME, the study population was necessarily heterogeneous. Surgery was performed wherever possible; definitive chemoradiotherapy was added as the preferred treatment modality for patients in whom complete resection was difficult to achieve.

In conclusion, in this study, pretreatment CAR was associated with OS and PFS in patients with locally advanced SCC of the EAC and ME. Pretreatment NLR was also associated with OS. No relationship was observed between tumor- or treatment-related factors and OS or PFS.
